# Green Turtle *Chelonia mydas* Spatial Use Within the Gorgona National Natural Park, Colombian Pacific: Implications for Local Conservation

**DOI:** 10.1002/ece3.73319

**Published:** 2026-06-02

**Authors:** Diego F. Amorocho, Andrew S. Maurer, Juan S. Ayala, Fernando Castro, Jeffrey A. Seminoff

**Affiliations:** ^1^ Centro de Investigación Para el Manejo Ambiental y el Desarrollo (CIMAD) Cali Colombia; ^2^ National Research Council Washington DC California USA; ^3^ NOAA, Marine Mammal and Turtle Division Southwest Fisheries Science Center La Jolla California USA; ^4^ Laboratorio de Herpetología, Departamento de Biología Universidad del Valle Cali Colombia

**Keywords:** Eastern Tropical Pacific, home range, marine protected area, satellite telemetry

## Abstract

Animal movement patterns over time and space underlie the design of effective protected areas (PAs). Gorgona National Natural Park (GNNP) is a PA in the Colombian Pacific for which evaluation of protections afforded to local conservation‐dependent species is needed. We address this need herein by assessing green turtle ( *Chelonia mydas* ) movement and residency patterns at GNNP. Satellite telemetry for 10 juvenile turtles tracked during 2009–2012 provided evidence that nine individuals exhibited residency behavior. Kernel utilization distributions suggested that the resident population's core 50% area of activity was fully contained within GNNP, while the 90% activity space was 89% contained. One turtle left the study area, migrating south. We offer these findings at a pivotal time, when plans for constructing a military complex within GNNP are underway. Our study highlights that GNNP protects critical habitats for imperiled species and underscores the need for thoughtful consideration of future developments near sensitive PAs.

## Graphical abstract

This study explores the spatial ecology of green turtles within the Gorgona National Natural Park (GNNP), providing insights into habitat preferences, movement patterns, and conservation implications for this vulnerable species in the Colombian Pacific. Ten green turtles were tracked in the GNNP using satellite telemetry, with tracking duration ranging from 10 to 170 Days. We found that the core area of activity was fully contained within GNP boundaries, while the broader activity space was 89% protected.

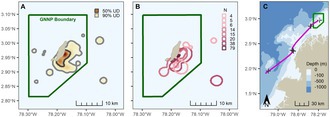

## 1 Introduction

The spatial‐use patterns of marine megafauna have direct implications for foraging ecology, growth, and survival. When considered in the context of existing human pressures, understanding the movement patterns of impacted species is essential for advancing conservation efforts (Baudouin et al. 2015; Allen and Singh 2016). Evidence‐based management tools such as time‐area closures and marine protected areas (MPAs) have been widely implemented and can be effective for protecting particular life‐history stages or reducing bycatch of nontarget species (Roberts et al. 2005; Evans 2018; Hays et al. 2019). However, their effectiveness is ultimately reliant on spatial data for local species (Chapman et al. 2005), which can be challenging to obtain for many taxa.

Green turtles ( *Chelonia mydas* ) exemplify the potential benefits from marine spatial planning, particularly when challenges to obtaining location data are overcome. Well‐documented fidelity of adults to nesting sites (Carr 1975; Mortimer and Portier 1989; Fitzsimmons et al. 1995) and coastal foraging areas (Broderick et al. 2007; Dutton et al. 2019) means that spatial protections can be effective over long‐term scales. However, turtle‐focused management is often hindered by limited information on local movements and aggregation sites (Santos et al. 2021). Simply put—sea turtles are difficult to observe and/or track in marine habitats. Moreover, existing data on sea turtle movement are disproportionately focused on adults, leaving a knowledge gap for immature stages that have been shown to be key to population growth in some contexts (Wildermann et al. 2018).

Here, we focus on a green turtle foraging aggregation in the Eastern Tropical Pacific (ETP), a region where the species is widespread, foraging and nesting in all countries from Mexico to Peru. Numerous MPAs and special management areas are present in the ETP, which collectively provide protection for all life stages of green turtles. In the easternmost region of the ETP lies Gorgona National Natural Park (GNNP), located ca. 30 km off the coast of Colombia. This park includes Gorgona and Gorgonilla Islands and surrounding marine habitats that are known to be used by green turtles, and less frequently hawksbills ( *Eretmochelys imbricata* , Amorocho and Tobón 2012). Previous research described the nesting origins (Amorocho et al. 2012), demographic structure (Sampson et al. 2014, 2015), and foraging ecology (Amorocho and Reina 2007; Sampson et al. 2018) of resident green turtles, but to date there have been no descriptions of in‐water movement.

Data on green turtle movements and residency patterns within GNNP would be beneficial in the short term to inform management related to the construction of a Coast Guard Substation by the Colombian Navy. Started in 2023, this project has faced significant criticism (CIRPA 2023). A scientific oversight group—the Gorgona Scientific Committee—raised several concerns revolving around (1) the absence of comprehensive prior research; (2) an insufficiently detailed and transparent management plan; and (3) the impacts resulting from the construction of a 180‐m pier, a radar installation, and a 7000‐gal fuel tank on the shore (ANLA 2023; TCPS 2024). In GNNP, green turtles are thought to primarily use the eastern side of Gorgona Island due to the presence of coral reefs (Sampson et al. 2014), and the same area of coastline is designated for the 180‐m pier.

We use satellite telemetry to assess green turtle movement patterns in and around GNNP. Our objectives were to (1) evaluate whether high‐use areas overlap with park boundaries; and (2) characterize residency patterns. This knowledge can inform the management of GNNP for the benefit of an important population of juvenile green turtles in the ETP, simultaneously helping to clarify the role of this insular foraging area in the life history of green turtles in the region.

## 2 Materials and Methods

### 2.1 Study Area

**FIGURE 1:**
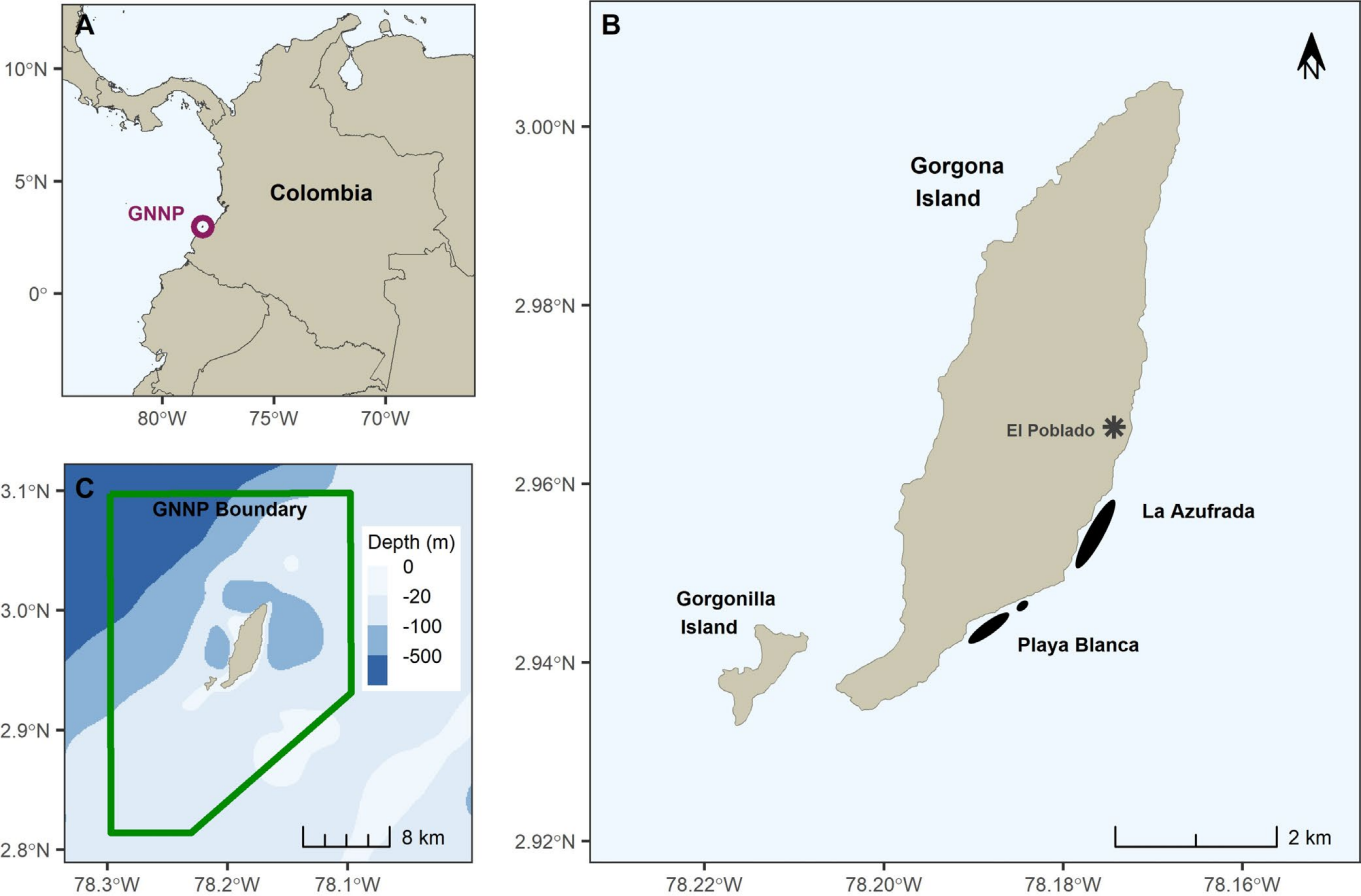
(A) Location of Gorgona National Natural Park (GNNP) in the Colombian Pacific. (B) Locations for turtle capture sites (Playa Blanca and La Azufrada reefs; black polygons) and the El Poblado population center on the island. (C) The GNNP boundary with relative bathymetry.

This study was carried out from 2009 to 2012 in GNNP (02°49′–03°06′N, 78°06′–78°18′W; Figure 1). GNNP was established in 1984 after serving as the site of a high‐security prison during 1959–1984, and encompasses 620 km^2^ of marine waters, with the 13.3‐km^2^ Gorgona Island centrally located within park boundaries; the smaller Gorgonilla Island is situated to the southwest. Soft bottoms, rocky coastlines, sandy beaches, coral reefs, and soft coral areas make up the marine and coastal ecosystems of GNNP; no seagrasses are present. A 270‐m deep underwater canyon separates GNNP from mainland Colombia, potentially influencing the movement and exchange of marine fauna.

Green turtle capture efforts were carried out at two fringing reefs located along the southeastern side of Gorgona Island—La Azufrada (0.112 km^2^) and Playa Blanca (0.109 km^2^). These reefs are dominated by the scleractinian corals *Pocillopora damicornis* and *P. elegans*, with adjacent sandy‐bottom areas at ca. 15‐m depth (Zapata and Vargas‐Ángel 2003). Coral and algal cover at these sites are dynamic; fluctuations in cover at La Azufrada are influenced by sedimentation and stress, with previous estimates of coral cover ranging 55%–65% and algal cover ranging 29%–37% (Zapata et al. 2010). A total of 85 species of algae has been identified from the coastal waters of Gorgona (Bula‐Meyer 1995; Giraldo et al. 2014). A study carried out in 2010–2011 reported that macroalgae species were comprised of 55.8% *Rhodophyta* (i.e., red algae), 27.9% *Chlorophyta* (green algae), and 14% *Heterokontophyta* (golden and brown algae; Murillo Muñoz and Peña Salamanca 2014).

### 2.2 Turtle Capture and Transmitter Deployment

Green turtles were hand‐captured by snorkelers at La Azufrada and Playa Blanca reefs between 20:00 and 21:00 h following Amorocho and Reina (2007). Captured turtles were taken to shore where satellite transmitters were attached and measurements were taken including curved carapace length (CCL; ±0.5 cm) and weight (±0.5 kg). Transmitters were affixed to the highest point of each turtle's carapace using a quick‐setting epoxy (Anchorfix II, Sika) that hardened within ~30 min. To aid transmitter retention, shell surfaces were treated with sandpaper and an alcohol solution until clean of algae and other irregularities. All turtles were released from the shore adjacent to the reef within 2 h of capture.

Transmitters (either Wildlife Computers SPOT‐293A or Sirtrack SPOT 5) provided location data via the Argos system and were programmed to alternate between on and off status at 6‐h intervals. Argos fixes are categorized into one of seven location classes (LCs) representing estimated accuracy: 3, 2, 1, 0, A, B, and Z. Two filtering frameworks were imposed based on these LCs. First, for home‐range analyses (detailed below), 0, B, and Z fixes—associated with the highest error (Hays et al. 2001; Witt et al. 2010)—were excluded to reduce the potential for erroneous inferences within a fine‐scale working extent. Second, in the case of migratory behavior, only Z fixes were filtered to retain information on directional movement (within a broader extent over which more spatial error was tolerable).

### 2.3 Movement Analyses

Tracking data were used to identify areas of high use around GNNP for turtles that exhibited nonmigratory (i.e., residency) behavior. Utilization distributions (UDs) were estimated at the population and individual levels to represent turtle home ranges. Argos fixes are associated with error of sufficient scale that inference is constrained for finer‐scale measures of movement, especially given low sample sizes; thus, no measures of home range size/area are presented herein. Rather, our focus was to map the areas of relatively concentrated turtle activity.

UDs were generated with a weighted kernel density estimator via the R package “spatialEco” (Evans and Murphy 2023). A two‐part weight was assigned to each satellite fix to account for Argos error and within‐track autocorrelation. Weight for Argos error was proportional to the inverse of the mean metric error for each LC reported by Witt et al. (2010); autocorrelation weights were estimated via the autocorrelated kernel density estimator developed by Fleming and Calabrese (2023), entailing fitting a continuous‐time movement model to individual tracks before estimating a weight reflecting relative autocorrelation for each location; autocorrelation weights for an individual summed to one such that turtles had equal weight in population‐level analyses. The two weights were then multiplied to form a single weight for kernel density estimation. All UDs were estimated within a fixed, conservatively large extent (33.7 × 19.3 km). Bandwidths were selected via a version of the ad hoc approach (Kie 2013); specifically, 50% UDs were generated for groups of fixes (all nonmigratory individuals, plus a population‐level grouping) using bandwidths at decreasing 250‐m increments, where the final bandwidth before the UD polygon became non‐contiguous was selected. For two instances in which limited sample sizes prevented a non‐contiguous UD polygon within reasonable bandwidths, the smallest bandwidth resulting in two polygons was used. All individual 50% UDs are presented in the results, in addition to a population‐level kernel bounded by the non‐contiguous 90% UD contour.

Raw location data for a lone individual that exhibited a migration away from GNNP were analyzed with a correlated random walk model (Jonsen et al. 2005, 2023) applied frequently to Argos data depicting sea turtle migrations (e.g., Maurer, Seminoff, et al. 2024). This approach was used to estimate one “true” location per day, a time step selected given a mean raw fix interval of 1.3 days. Bathymetry data used in mapping were derived from the General Bathymetric Chart of the Oceans (GEBCO 2023).

## 3 Results

Ten juvenile green turtles (52.4–72.8 cm CCL) were equipped with transmitters during 4 years of sampling (Table S1). Transmitters provided 8–156 fixes for durations of 10–170 days, after which they stopped working due to presumed detachment or failure. Most fixes were LC B, with estimated error exceeding that useful for inference within the working extent of GNNP. Filtering resulted in the exclusion of 74% of raw fixes, leaving 195 locations (4–79 per turtle) for movement analyses.

**FIGURE 2:**
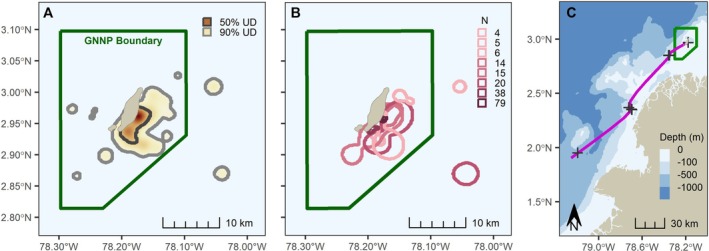
Space use of green turtles ( *Chelonia mydas* ) satellite‐tracked from Gorgona National Natural Park (GNNP), Colombian Pacific. (A) The combined space use pattern for nine turtles is depicted with a continuous kernel density estimate; darker orange‐red colors denote higher areas of use. (B) Individual 50% UDs are shown, with increasing sample sizes of satellite fixes (N) denoted with darker hue. (C) Longer‐range movements are mapped for a single nonresident individual that transited south from GNNP.

Nine turtles were identified as nonmigratory for the study period, remaining near GNNP while transmitters were functional (Figures 2, S1). Population‐ and individual‐level UDs (Figure 2A,B, respectively) suggested a defined, core activity center close to Gorgona Island's southeastern shoreline. Satellite fixes did reflect movement outside this core area, including to the western side of the island, but only rarely; the main activity space spanned the eastern side of the island, characterized by relatively shallow bathymetry, calm ocean conditions, and the aforementioned reef habitats. GNNP boundaries contained 100% of the population‐level 50% UD polygon and 89% of the broader 90% UD (both computed with a 3.25‐km bandwidth).

A single turtle—Turtle 10—exhibited migratory behavior, departing Gorgona Island on a southward heading 1–5 days after release (Figure 2C). Eight raw locations were collected for this individual over approximately 10 days (mean travel speed = 18.23 ± 4.37 km/d) with transmissions apparently ending mid‐migration. Although conjectural, it appeared to be on the verge of leaving Colombian waters for Ecuador or further south. The migration generally tracked relatively shallow water along the Colombian coast.

## 4 Discussion

Our study of green turtles within GNNP is the first to depict the movements and spatial ecology of green turtles in the Colombian Pacific. Satellite telemetry revealed that 9 of the 10 turtles remained near GNNP during their tracking periods, with core areas completely contained within park boundaries. More specifically, tracked individuals concentrated activity along the southeastern coast of Gorgona Island (Figure 2A), which is largely protected from prevailing westerly ocean swell and is the site of the La Azufrada and Playa Blanca Reefs. Although we acknowledge that this finding is influenced by capture bias, consistent fidelity to these reef habitats nonetheless provides compelling evidence of their value for local green turtles. Coral formations in this sector present a reef scaffold (Zapata et al. 2001; Zapata and Vargas‐Ángel 2003) that likely provides adequate shelter for local turtles as well as habitat structure for primary prey groups (Amorocho and Reina 2007). Put plainly, such results demonstrate that GNNP offers the necessary resources for local turtles. As such, future tourism and infrastructural development must account for the presence of vulnerable green turtles in areas designated for construction activities.

It is known that green turtles typically exhibit strong foraging site fidelity during juvenile life phases (e.g., Seminoff et al. 2002; Hart and Fujisaki 2010; Lamont et al. 2015). Thus, with the size range of individuals in this study (52.4–72.8 cm CCL) indicating all were juvenile, it is perhaps not surprising that most remained in the GNNP vicinity during tracking. Unfortunately, our results do not offer conclusive evidence specific to how long residency durations may last. Our inference was limited by short transmission durations typical of green turtles in the eastern Pacific, which lack a well‐developed keratin layer onto which transmitter adhesives can bind (Seminoff et al. 2002, 2008; Hart et al. 2015). Future efforts are merited using updated attachment techniques that promote longer retention (Hanna 2021; Hart et al. 2021; Maurer, Eguchi, et al. 2024). Even the maximum residency duration we documented via satellite telemetry (~170 days) should underrepresent true maximum residency. Indeed, Rodríguez‐Zuluaga and Amorocho (2006) report three juveniles recaptured at the Gorgona reefs after seven, 12, and 30 months of initial tagging. Moreover, Sampson et al. (2015) marked 995 GNNP green turtles and recaptured only 33, but time‐at‐large ranged from 0.57 to 5.89 years (median = 1.99 years). Our results, taken together with these studies, offer two primary conclusions on residency behavior. First, juvenile green turtles within GNNP appear to exhibit strong site fidelity reflective of resident behavior. Second, although some turtles reside at GNNP long term (> 5 years), shorter residency durations are likely more common, as demonstrated by recaptures in other studies and the turtle we observed depart GNNP.

The individual that departed the study area last transmitted ~150 km south of GNNP. Its track, while incomplete, was directed toward continental foraging areas in Ecuador and Peru, where numerous green turtle foraging hotspots exist (Chaves et al. 2017; Jiménez et al. 2017). We postulate that its long‐distance movements were triggered by an ontogenic habitat shift (e.g., Hamabata et al. 2015), which have been reported for green turtles in continental habitats of Peru (Quiñones et al. 2022). To our knowledge, this is the first potential indication of an ontogenic shift involving an oceanic island in the ETP. Additional tracking efforts at Gorgona, as well as the nearby Galapagos Archipelago (Ecuador) and Cocos Island (Costa Rica), may shed more light on site connectivity and the influence of ontogeny on habitat use.

### 4.1 Conservation Implications

Our study provides actionable information for habitat managers at a pivotal time within the region. Within a broader context, our results combine with previous work to demonstrate that turtles exhibit temporary residency and leave GNNP, establishing connectivity with other sites (e.g., Amorocho et al. 2012). As such, local conservation efforts would ideally work in concert with actions in other jurisdictions with established movement connectivity, including continental Colombia and other areas in the ETP. At the local scale of GNNP, our data provide evidence that GNNP is effective in its function as a protected area; the park boundary contained the entire estimated population‐level core area (50% UD) and 89% of the 90% UD. We therefore recommend that park authorities maintain and intensify current patrols, surveillance, and control operations to enforce the ban on fishing gear within the MPA, rapidly detect and remove illegal nets or lines, and monitor habitat conditions. Furthermore, the park would benefit from continuing to forge collaborative agreements with local fishing communities, including workshops on bycatch reduction, participatory monitoring programs in fishing areas, and community‐led stewardship initiatives that promote turtle‐friendly fishing practices.

These findings also are highly relevant in the context of relatively new plans for Gorgona Island calling for expanded tourism and military infrastructure (Quartucci 2024; TCPS 2024), plus the construction of a large‐scale pier in areas adjacent to Gorgona's coral reef system (ANLA 2023). Thus, although green turtles have been shown to adapt to anthropogenic development in some cases (Mullaney et al. 2024), it is very likely that human impacts will grow substantially within the GNNP, including habitat degradation, acoustic disturbances, increased boat traffic (Tyson et al. 2017; Winkler et al. 2020), presenting long‐term risks to local flora and fauna. Direct impacts on turtles may increase, ranging from sublethal (e.g., effects of snorkelers; Griffin et al. 2017) to lethal (e.g., boat strikes; Lester et al. 2013). Accordingly, the conservation of the local green turtle population would benefit from increased efforts to monitor turtles and the local reef system (e.g., ANLA 2023; Mejía‐Renteria et al. 2024). Further, we suggest that management zoning aimed at limiting impacts from boat strikes and other human activities should be strongly considered (Shimada et al. 2017; Fuentes et al. 2021).

## Author Contributions

**Diego F. Amorocho:** conceptualization (lead), data curation (equal), funding acquisition (equal), investigation (lead), project administration (lead), resources (supporting), writing – original draft (equal), writing – review and editing (equal). **Andrew S. Maurer:** data curation (equal), formal analysis (lead), software (lead), writing – original draft (equal), writing – review and editing (equal). **Juan S. Ayala:** conceptualization (equal), data curation (supporting), investigation (supporting), writing – review and editing (supporting). **Fernando Castro:** conceptualization (equal), funding acquisition (supporting), project administration (equal), resources (equal), writing – review and editing (supporting). **Jeffrey A. Seminoff:** conceptualization (supporting), formal analysis (supporting), resources (equal), supervision (equal), writing – original draft (equal), writing – review and editing (equal).

## Conflicts of Interest

The authors declare no conflicts of interest.

## Supporting information


**Table S1:** Summary of green turtles ( *Chelonia mydas* ) tracked in Gorgona National Natural Park. Curved carapace length (CCL) is presented in cm. Tracking durations, rounded to the nearest whole day, are presented for data pre‐filtering. Location classes (LC) represent Argos spatial error estimates, and are listed in order of accuracy (3–Z).
**Figure S1:** Maps of filtered locations for all nonmigratory green turtles. Crosses are colored in blue hues unique to Argos location classes (LCs; representing estimated satellite fix error), with darker hues representing higher accuracy. Sequential locations are connected by green lines (but may entail long time steps). Raw data for the migratory Turtle 10 are shown in Figure 2C.

## Data Availability

The data that support the findings of this study are available in Dryad via the following link: 10.5061/dryad.8gtht773c.
