# Supplementary material for: Green Turtle Chelonia mydas Spatial Use Within the Gorgona National Natural Park, Colombian Pacific: Implications for Local Conservation
Source: Ecol Evol. 2026 Jun 2;16(6):e73319. doi: 10.1002/ece3.73319 (PMC13239815; doi:10.1002/ece3.73319)
Supplement: Supplementary file 1 — Table S1: Summary of green turtles ( Chelonia mydas ) tracked in Gorgona National Natural Park. Curved carapace length (CCL) is presented in cm. Tracking durations, rounded to the nearest whole day, are presented for data pre‐filtering. Location classes (LC) represent Argos spatial error estimates, and are listed in order of accuracy (3–Z). Figure S1: Maps of filtered locations for all nonmigratory green turtles. Crosses are colored in blue hues unique to Argos location classes (LCs; representing estimated satellite fix error), with darker hues representing higher accuracy. Sequential locations are connected by green lines (but may entail long time steps). Raw data for the migratory Turtle 10 are shown in Figure 2C. [file ECE3-16-e73319-s001.docx]

**Supporting Information**

Table S1. Summary of green turtles (*Chelonia mydas*) tracked in Gorgona National Natural Park. Curved carapace length (CCL) is presented in cm. Tracking durations, rounded to the nearest whole day, are presented for data pre-filtering. Location classes (LC) represent Argos spatial error estimates, and are listed in order of accuracy (3–Z).

| ID | CCL (cm) | Release | Duration (d) | Raw fixes | Filt. fixes | LC3 | LC2 | LC1 | LC0 | LCA | LCB | LCZ |
| --- | --- | --- | --- | --- | --- | --- | --- | --- | --- | --- | --- | --- |
| 1 | 62.0 | 26 Oct 2009 | 43 | 46 | 14 | 0 | 2 | 1 | 3 | 11 | 29 | 0 |
| 2 | 62.0 | 27 Oct 2009 | 170 | 156 | 79 | 19 | 21 | 6 | 6 | 33 | 70 | 1 |
| 3 | 68.5 | 27 Oct 2009 | 27 | 21 | 6 | 1 | 0 | 1 | 1 | 4 | 14 | 0 |
| 4 | 69.6 | 27 Oct 2009 | 44 | 58 | 20 | 1 | 4 | 4 | 4 | 11 | 34 | 0 |
| 5 | 72.8 | 27 Aug 2010 | 38 | 29 | 15 | 1 | 8 | 4 | 0 | 2 | 14 | 0 |
| 6 | 54.0 | 30 Jul 2010 | 22 | 16 | 4 | 1 | 1 | 0 | 0 | 2 | 12 | 0 |
| 7 | 52.4 | 15 Jul 2011 | 30 | 53 | 5 | 0 | 0 | 2 | 0 | 3 | 48 | 0 |
| 8 | 69.5 | 19 Aug 2011 | 34 | 54 | 6 | 2 | 0 | 1 | 0 | 3 | 48 | 0 |
| 9 | 72.4 | 14 Jul 2011 | 58 | 102 | 38 | 13 | 12 | 2 | 0 | 11 | 64 | 0 |
| 10 | 61.0 | 21 Jun 2012 | 10 | 8 | 8 | 0 | 0 | 1 | 0 | 4 | 3 | 0 |

^a^ Turtle 10 was the lone individual that migrated away from GNNP (Figure 2C), entailing distinct filtering guidelines


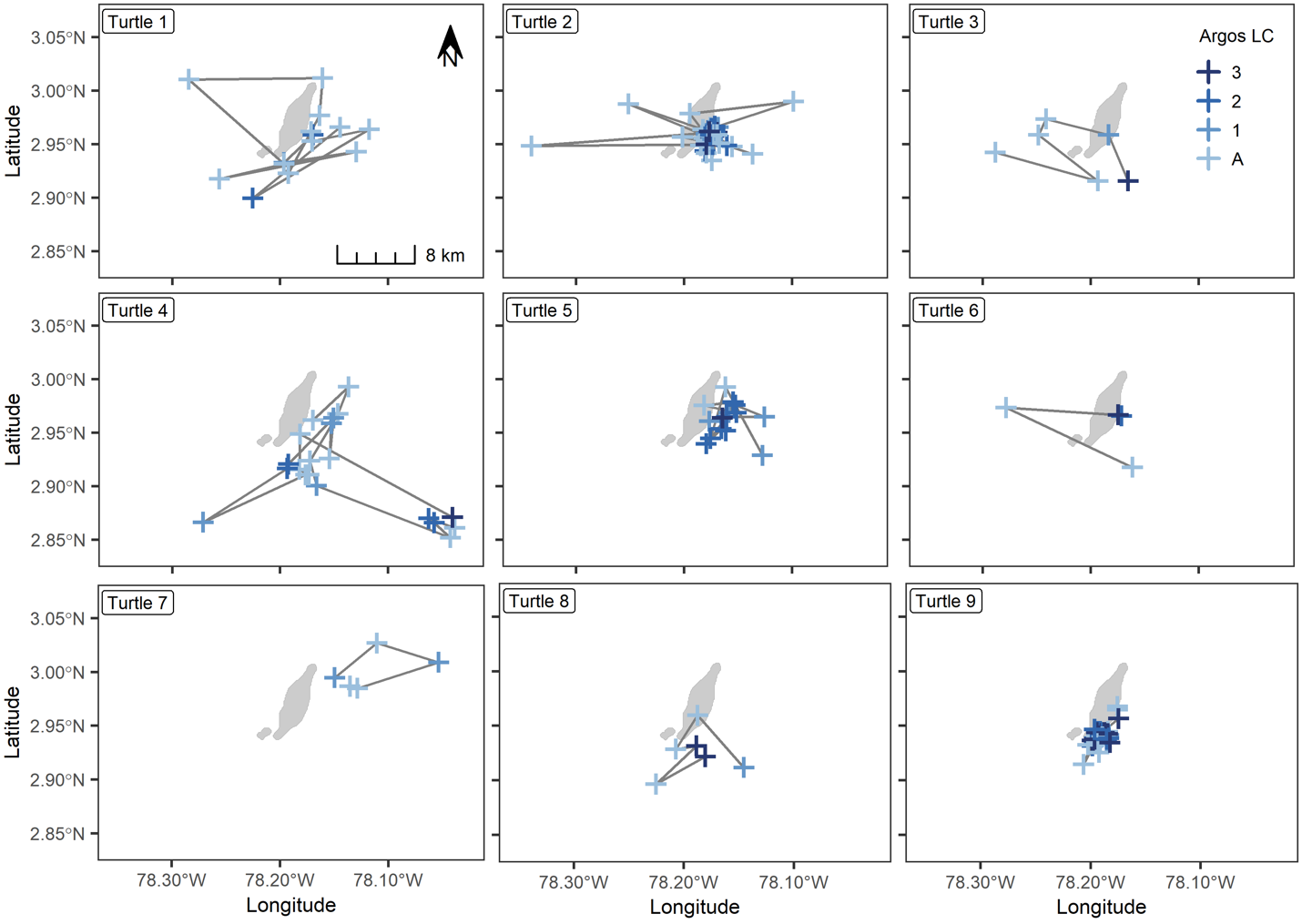


Figure S1. Maps of filtered locations for all nonmigratory green turtles. Crosses are colored in blue hues unique to Argos location classes (LCs; representing estimated satellite fix error), with darker hues representing higher accuracy. Sequential locations are connected by green lines (but may entail long time steps). Raw data for the migratory Turtle 10 are shown in Figure 2C.
